# Effects of family conversation on health care practices in Ethiopia: a propensity score matched analysis

**DOI:** 10.1186/s12884-018-1978-8

**Published:** 2018-09-24

**Authors:** Dessalew Emaway Altaye, Ali Mehryar Karim, Wuleta Betemariam, Nebreed Fesseha Zemichael, Tesfaye Shigute, Pauline Scheelbeek

**Affiliations:** 1 The Last Ten Kilometers Project (L10K) 2020, JSI Research & Training Institute, Inc., Bole Sub-City, Kebele 03/05, Hs #, 2111 Addis Ababa, Ethiopia; 2 JSI Research & Training Institute, Inc., 1616 N Fort Myer Dr, 16th Floor, Arlington, VA 22209 USA; 3 Department of Epidemiology and Population Health, London School of Hygiene & Tropical Medicine, London, UK

**Keywords:** Maternal, Newborn, Family conversation, Outcomes, Ethiopia

## Abstract

**Background:**

Maternal and newborn mortality rates in Ethiopia are among the highest in sub-Saharan Africa. The majority of deaths take place during childbirth or within the following 48 h. Therefore, ensuring facility deliveries with emergency obstetric and newborn care services available and immediate postnatal follow-up are key strategies to increase survival. In early 2014, the Family Conversation was implemented in 115 rural districts in Ethiopia, covering about 17 million people. It aimed to reduce maternal and newborn mortality by promoting institutional delivery, early postnatal care and immediate newborn care practices. More than 6000 Health Extension Workers were trained to initiate home-based Family Conversations with pregnant women and key household decision-makers. These conversations included discussions on birth preparedness, postpartum and newborn care needs to engage key household stakeholders in supporting women during their pregnancy, labor and postpartum periods. This paper examines the effects of the Family Conversation strategy on maternal and neonatal care practices.

**Methods:**

We used cross-sectional data from a representative sample of 4684 women with children aged 0–11 months from 115 districts collected between December 2014 and January 2015. We compared intrapartum and newborn care practices related to the most recent childbirth, between those who reported having participated in a Family Conversation during pregnancy, and those who had not. Propensity score matched analysis was used to estimate average treatment effects of the Family Conversation strategy on intrapartum and newborn care practices, including institutional delivery, early postnatal and immediate breastfeeding.

**Results:**

About 17% of the respondents reported having had a Family Conversation during their last pregnancy. Average treatment effects of 7, 12, 9 and 16 percentage-points respectively were found for institutional deliveries, early postnatal care, clean cord care and thermal care of the newborn (*p* < 0.05).

**Conclusion:**

We found evidence that Family Conversation, and specifically the involvement of household members who were major decision-makers, was associated with better intrapartum and newborn care practices. This study adds to the evidence base that involving husbands and mothers-in-law, as well as pregnant women, in behavior change communication interventions could be critical for improving maternal and newborn care and therewith lowering mortality rates.

**Electronic supplementary material:**

The online version of this article (10.1186/s12884-018-1978-8) contains supplementary material, which is available to authorized users.

## Background

Since 2000, Ethiopian health services have put in tremendous efforts to reduce maternal, neonatal and under-5 mortality. The achievements so far vary quite substantially: despite a reduction in the maternal mortality ratio from 871 deaths per 100,000 live births in 2000, to 412 deaths per 100,000 live births in 2016 [1, 2], maternal mortality in Ethiopia remains amongst the highest in sub-Saharan Africa. A substantial reduction was reported in the under-five mortality rate, which came down from 188 deaths per 1000 live births in 2000, to 67 deaths per 1000 live births in 2016. However, in the same period, the neonatal mortality rate came down at a slower pace from 49 deaths per 1000 live births in 2000, to 29 deaths per 1000 live births in 2016 and thus the contribution of neonatal deaths to under-five deaths increased from 26% in 2000, to 43% in 2016 [2]. Like other low and middle-income countries, the majority of neonatal and maternal deaths take place at childbirth or within the first 48 h after birth [3, 4].

Ensuring childbirth takes place at institutions with emergency obstetric and newborn care services, as well as postnatal follow-up of mother and newborn within 48 h of delivery, has been shown to reduce maternal and neonatal mortality [5–8]. Worldwide, both strategies have been adopted as mainstream approaches to reduce maternal and newborn deaths [9].

In Ethiopia, the coverage of services for mothers and newborns at critical times has been relatively low; however, some improvements have been reported in the past 5 years. The Ethiopian Demographic and Health Survey reported in 2011 that institutional delivery occurred for 10% of all deliveries, while 7% of mothers and newborns received early postnatal care; in 2016 this had increased to 26% and 16%, respectively [2]. In 2010, a large population-based survey in Ethiopia on household level newborn care practices showed that only 25% of newborns got thermal care, 46% had clean cord care and 54% of babies were put to breast immediately after birth [10].

The power structures within families has been proposed as one reason why institutional delivery rates and postnatal care coverage are so low [11, 12]. In Ethiopia, a relatively traditional society, family power structures can have a negative effect on health outcomes [13]. It often occurs, for example, that a pregnant woman – who has been trained by health workers and is aware of her need for care – is not the person who decides whether to go to the health facility; her husband, her mother-in-law, her mother and sometimes her neighbors – who might be less informed about the most up-to-date care recommendations and might therefore be inclined to follow more traditional practices [14, 15] – take decisions regarding household practices and care seeking on her behalf [16–18].

The overriding factors that affect decision making against the “preferable practices” as promoted by health workers, can be found in the lack of awareness of obstetrical risks in the absence of skilled personal and equipment, in combination with deep rooted cultural beliefs and practices, which include a traditional preference for home delivery [11], discarding colostrum, delaying breastfeeding, pre-lacteal feeding, applying something to the umbilical cord, insufficient thermal care, early bathing of the newborn and low care seeking for MNH [19, 20]. Therefore, successful engagements of the community health workers with family members and primary care givers for pregnant women and newborns and adequate communication of the evidence and risks around child birth for both mother and newborn could facilitate improved health care-seeking, as well as improved uptake of several MNH care practices and behaviors. Community health workers who are deployed at the grassroots level, could be well positioned health cadres to discuss deep rooted religious and cultural practices that are risky to newborn and maternal health [21, 22].

This paper reports on an evaluation of the association between and possible effect of Family Conversations on MNH care seeking and newborn care practices. It is one in a series of four papers investigating innovations designed to support Ethiopia’s flagship Health Extension Program (HEP) to improve MNH in Ethiopia. The other three papers consider the Women’s Development Army, Community-Based Data for Decision-Making (CBDDM) and a Participatory Community Quality Improvement strategy [23–25].

## Methods

### Study setting

The study setting has been described in detail in Additional files 1, 2 and 3 of the first paper in this supplement [23]. In short, this study was conducted in 115 rural districts from four agrarian regions (Tigray, Amhara, Oromia and Southern Nations, Nationalities and Peoples region) of Ethiopia.

The primary health care system of Ethiopia, the foundation of the three-tier health system of the country, comprises of a district hospital, three to four health centers, each serving about five health posts. The primary health care unit (PHCU), formed of one health center and five satellite health posts, serves an average of 25,000 people. Health center staff provide basic preventive and curative health services including basic emergency obstetric and newborn care (BEmONC), and supervise the satellite health posts. The Health Extension Program, Ethiopia’s flagship program launched in 2004, comprises the health posts with two female Health Extension Workers (HEWs) serving a community (*kebele*) of about five thousand people with basic community-based promotive, preventive and curative health services. To further extend the reach of the Health Extension Program and mobilize the community and households, a network of Women’s Development Army (WDA) volunteers, previously called Health Development Army, was established in 2011. It comprises one WDA member for every five households based on social and geographical proximity, whose role is to influence community members to practice healthy behaviors. For every six WDA members, and thus for every 30 households, one WDA member was chosen as a ‘1-to-30’ team leader.

The Last Ten Kilometers (L10 K) Project has been working closely with the government to implement innovative strategies to engage local communities in improving reproductive, maternal, newborn and child health interventions. L10 K aimed to contribute towards the achievement of the Millennium Development Goal targets related to maternal and child health in Ethiopia through enhancing interaction between households, communities and the Health Extension Program. L10 K’s foundational community strategy, referred to as the L10 K Platform, was implemented in 115 *woredas* (districts) of the four regions. This Platform includes community level innovations such as Community Based Data for Decision Making (CBDDM), Family Conversation and Birth Notification. CBDDM is a surveillance mechanism which enables WDA members to map their network of 25 to 30 households to ensure needed and targeted health services to households. One of L10 K’s interventions to improve demand for and utilization of maternal and neonatal health care, and in particular, issues related to family power structures, is the Family Conversation. A Family Conversation is a health education session organized by a HEW (usually together with a WDA team leader) at a pregnant woman’s home.

### Description of the family conversation

The Family Conversation is an informal meeting between a health worker and several household and community members who potentially play a role in the decision making around a pregnant woman and/or her newborn. The conversation was designed to engage close family members and relatives of the pregnant woman (such as her husband, her mother, mother in-law, elder sisters) who are expected to support her during pregnancy, labor, delivery and the postpartum period. It creates an opportunity to discuss issues such as birth preparedness and essential newborn practices with all these family members together. The conversation consists of two parts: an early visit when a woman is 4–6 months pregnant and a later visit when she is around 8–9 months pregnant. For the first visit, once a pregnant woman has been identified by the WDA or a HEW, a convenient moment is sought to get the family together. In the meeting, the HEW or WDA member, and sometimes both HEWs and WDA members together, provide education on favorable key actions by the household during pregnancy and in the first months of the newborn’s life to minimize risks to mother and child. An important topic that is addressed in the meeting is the benefit of continued antenatal care for both mother and child. Families are encouraged to engage in antenatal care, including at least one visit taking place at the health center, and are talked through the appropriate timing of antenatal care. Other topics discussed during this meeting include the importance of good nutrition, the need to start saving money for the delivery, knowledge about danger signs during pregnancy and how to act if an emergency occurs. The HEW or WDA member discusses potential barriers to care with the families and they try to determine solutions jointly to overcome these barriers. The pregnant woman is given a family health guide as a reference booklet: this guide contains illustrated educational material around antenatal care, birth preparedness, delivery, postnatal care, danger signs, and early newborn care.

When the pregnant woman approaches delivery, the HEW or WDA member organizes a second Family Conversation session. The main aim of this session is to develop a plan for labor and delivery. The HEW or WDA member will reiterate how to recognize labor and any danger signs and help the families develop a clear birth plan. This plan covers decisions on issues such as the essential items the family need to have ready when labor starts, the place of delivery, what transport will be used, how costs for transport and other services will be covered, who will support the pregnant woman during labor and delivery, and who will take care of the household, and possibly other children, whilst the pregnant woman is away. If the HEW and WDAs notice any potential barrier in the family for the intended behavior, they may arrange extra visits to the family to conduct further dialogues or reinforce messages with family members. In addition, families are educated using the family health guide on immediate newborn care practices such as clean cord care, thermal care, and immediate and exclusive breastfeeding.

The Family Conversation strategy was implemented in 3070 kebeles, and over 6000 HEWs were given a two-day orientation training on Family Conversations, integrated with the woreda level performance review and a refresher training meeting, between late 2013 and early 2014. The HEWs were oriented at woreda level in 115 woredas by field-level project staff. The orientation included a presentation on the benefit of engaging husbands and families for reproductive, maternal, newborn and child health care, discussions on how to use the Family Conversation guides and the family health guide, role play, and how HEWs can orient the WDA at village level. HEWs were also oriented on how to provide on the job training and mentoring to the WDA while they conduct a Family Conversation. After the orientation, supportive supervision was conducted with HEWs by health center, woreda and project staff. Performance review and refresher training meetings were organized after 4 months of implementation. Implementation status was followed through the supportive supervision and the project management information system. The evaluation survey was conducted between December 2014 and January 2015.

### Study participants

The study participants were women with children aged 0–11 months.

### Study design

This was a cross-sectional study that measured the effects of self-reported exposure to the Family Conversation strategy during antenatal period on maternal and newborn health care practices during childbirth and postpartum period.

### Sample size

This study analysed follow-up data from before-and-after household and health post surveys conducted in January 2010–February 2011 and January 2014–February 2015 to evaluate the L10 K program. In the follow-up survey 4801 new mothers were interviewed from 400 kebeles representing all 115 L10 K intervention woredas. Data from 10 kebeles were dropped, as the kebele level information of some of the contextual variables were not available. Thus, the sample size available for this study was 4684 women with children aged 0 to 11 months (see Additional file 1 of the first paper in this supplement for the sample size calculation for the evaluation [23]).

### Data collection

Ethical clearance was obtained from the Institutional Review Boards of the respective Regional Health Bureaus and that of JSI Research & Training Institute, Inc. The survey methodology is described in detail in the Additional files 1, 2 and 3 of the first paper in this supplement [23]. In brief, two-stage stratified cluster sampling representing the L10 K platform woredas was performed to draw a representative sample of households with children aged 0–11 months. At the first stage, kebeles were selected as primary sampling units with probability proportional to their estimated population sizes, stratified by region. At the second stage, the sampling strategy described by Lemeshow and Robinson (1985) was used to select households with target respondents [26]. Accordingly, the first household was selected randomly from the middle of the kebele and then every fifth household was visited, moving away from the middle, and if the household had women with children aged 0–11 months, they were interviewed, after seeking their consent. If the respondent was under 18 years old, then consent was sought from her husband, parents or guardian. A quota of 12 women was interviewed from each kebele to obtain information on their socio-demographic background and the MNH care behavior and practices associated with their most recent pregnancy and childbirth. In a very few cases the interviewers mistakenly interviewed a few more or a few less women than the quota for a kebele.

The health posts for the sampled kebeles were visited to conduct interviews with the HEWs and review the health post records to obtain information on the HEW-population ratio, the WDA team leader-household ratio, and the intensity of the CBDDM activities of the L10 K project [24].

### Outcome measurements

In this study five MNH care practices before, during and after childbirth were considered as program outcomes. The definitions of each of the outcomes of interest are shown in Table 1.

**Table 1:** Definition of maternal and newborn care indicators

Indicator	Definition
Delivery at health facility	The percentage of women with children aged 0–11 months whose last childbirth was at a health facility
Early postnatal care	The percentage of women with children aged 0–11 months who were visited by HEWs at home for postnatal care or newborn care within 48 h of last childbirth
Clean cord care of their newborn	The percent of most recent births, not assisted by skilled birth attendants, among women with children aged 0–11 months whose umbilical cord was cut with a sterile instrument, tied with sterile thread, and had nothing appliedto the cut end of the umbilical cord
Thermal care of their newborn	The percent of women with children aged 0–11 months who were not assisted by skilled professional but who dried and wrapped their last newborn immediatelyafter birth, delayed bathing the newborn by 6 h or more, and always maintained skin-to-skin contact with the baby
Immediate breastfeeding	The percentage of women who initiated breastfeeding the newborn immediately after birth, among those who were not assisted by skilled birth attendants

*HEW* Health Extension Worker

### Independent variables

We hypothesized that the selected maternal and newborn health care practices would be influenced by the Family Conversations. The exposure in our study was therefore defined as self-reported participation in a Family Conversation during the most recent pregnancy. Respondents were considered to have been exposed to a Family Conversation if they reported that the HEW or the WDA member had had at least one meeting with the pregnant woman and her family to discuss pregnancy care and childbirth issues.

Data on possible confounders were also captured and included individual, household and kebele-level sample characteristics, administrative regions, and antenatal care attendance by the respondents. The individual level characteristics considered were age, education, marital status, parity and religion; the household-level characteristics were wealth and distance of the respondents’ household from the nearest health facility; and the kebele-level characteristics considered were HEW to population ratio*,* the WDA density and the intensity of CBDDM implementation. In addition, receiving early postnatal care was also used as a control variable for the intervention effects on newborn care practices.

A wealth index score was constructed for each household with the principal component analysis of the household possessions (electricity, watch, radio, television, mobile phone, telephone, refrigerator, table, chair, bed, electric stove and kerosene lamp), and household characteristics (type of latrine and water source). Subsequently, households were ranked according to wealth score and then divided into five quintiles [27].

The WDA density in a kebele was the ratio between the total number of households and the number of active WDA team leaders in that kebele. Active WDA team leaders were those who met with the HEW and had discussions on Health Extension Program issues during the 3 months preceding the survey.

Each kebele was assigned an intensity of CBDDM implementation score based four items obtained from HEW interviews and health post records: 1) proportion of WDA team leaders in the kebele who have a surveillance map of their neighborhood; 2) proportion of WDA team leaders who reported surveillance data to a HEW, or the HEW had collected it from the WDA team leader during last 3 months; 3) whether or not the HEW had updated health post records with surveillance data; and 4) whether the kebele leaders used surveillance data for monitoring the utilization of maternal and newborn health services during last 3 months. All four items were given equal weights and the score was re-scaled to range between 0 and 10 and the Cronbach’s alpha of the index was 0.75 [24].

### Data analysis

First, the differentials in the individual, household and kebele-level sample characteristics, administrative regions and antenatal care attendance by the respondents according to exposure to Family Conversation strategy were assessed using Pearson’s chi-squared statistics adjusted for survey design. Stata 14.2 was used for the analysis [28].

Average treatment effect of exposure to Family Conversations on selected perinatal and postnatal maternal and newborn health care practice indicators were estimated using propensity score matching (PSM). The technique imputes missing potential outcome for each participant by using an average of the outcomes of similar participants that receive the other treatment level. Similarities between exposed and unexposed respondents were based on the probabilities of being exposed, known as the propensity score. Logit models were used to estimate the propensity scores for exposure to the Family Conversation strategy with individual, household and kebele*-*level sample characteristics and antenatal care attendance as the predictors. One model was estimated for the sample that included all deliveries and another model for the sample that included the deliveries that were not attended by skilled health professionals. The average treatment effect was then calculated by taking the average of the difference between the observed and potential outcomes for each participant. We assessed average treatment effects of exposure to Family Conversation on the outcomes of interest using Stata’s ‘teffects psmatch’ procedure [29].

To assess the adequacy of the matching, we assessed the balance of covariates (the predictors of the logit model) across exposed and non-exposed groups after matching. Balance was considered adequate if the standardized differences of the covariates between the two groups were less than 10% after matching [30]. A minimum of one-to-one match per participant was considered adequate if the balancing property was satisfied. If a one-to-one match did not satisfy the balancing property, then the minimum number of matches per participant was incrementally increased until the balancing property was satisfied [30, 31]. The method selected an extra match per participant if propensity score was tied. Average treatment effects are presented from PSM models that satisfied the balancing property.

## Results

About 17% of the sample reported that they had had a Family Conversation during their most recent pregnancy. Of those who had a Family Conversation, nearly three-quarters had had at least two sessions and one third of them had had three or more sessions. In the majority of cases the husband (80%) participated in the Family Conversation followed by the woman’s mother (27%), mother-in-law (19%), neighbor (16%), and sister (7%), as well as other people. Of those who reported having had Family Conversation, more than two-thirds were conducted by HEWs, with and without WDA members, and about a quarter of the sessions were conducted by WDA members only.

Table 2 presents the background characteristics of the study participants (women with children aged 0–11 months) stratified by exposure to Family Conversations during their most recent pregnancy. We found evidence of differences in exposure to Family Conversations by age, education, parity, religion, administrative region, WDA density of the kebele, intensity of CBDDM implementation score and number of antenatal care visits (*p* < 0.05). Women who were more literate, who were older, those who were living in a higher CBDDM score area, who were living in Tigray region, who followed Orthodox religion and those who had more antenatal visits, were more likely to report that they had had a Family Conversation.

**Table 2:** Respondents’ characteristics by exposure status to Family Conversation, full sample

Sample characteristics	Category	Family conversation during last pregnancy					Total (*N* = 4684)	
		Not-exposed (*N* = 3926)		Exposed (*N* = 758)		*p*-value		
		*N*	%	*N*	%		*N*	%
Age group	15–19	361	9	34	5	0.005	395	8
	20–24	1068	27	190	25		1258	27
	25–34	1857	47	404	53		2261	48
	35–49	640	16	130	17		770	16
Education	Cannot read	2312	59	390	51	0.007	2702	58
	Primary	899	23	194	26		1093	23
	Higher	715	18	174	23		889	19
Marital status	Other	126	3	18	2	0.328	144	3
	Married/in union	3800	97	740	98		4540	97
Number of children	1	1111	28	165	22	0.020	1276	27
	2	671	17	127	17		799	17
	3	557	14	121	16		679	15
	4+	1586	40	344	45		1930	41
Religion	Orthodox	2108	54	502	66	0.016	2610	56
	Protestant	821	21	85	11		905	19
	Muslim	958	24	168	22		1125	24
	Other	35	1	4	1		39	1
Wealth quintile	Lowest	785	20	142	19	0.387	927	20
	Second	718	18	171	23		890	19
	Middle	769	20	144	19		914	20
	Fourth	817	21	138	18		955	20
	Highest	840	21	164	22		1004	21
Distance to any health Facility	< 30 min	2022	52	413	55	0.367	2435	52
	30 to 59 min	1225	31	243	32		1468	31
	1+ hours	679	17	102	13		781	17
Region	Tigray	444	11	179	24	< 0.001	623	13
	Amhara	1241	32	252	33		1492	32
	Oromia	1052	27	123	16		1175	25
	SNNP	1186	30	205	27		1390	30
HEW density (population per HEW in kebele)	2499	1747	45	311	41	0.582	2058	44
	2500 to 3499	1056	27	230	30		1287	28
	3500 to 4999	601	15	132	17		733	16
	5000+	522	13	85	11		607	13
WDA density	1 per 40 households	974	25	233	31	0.039	1206	26
	1 per 41–60 households	1602	41	340	45		1942	42
	1 per 61+ households	1354	35	185	24		1539	33
Intensity of CBDDM	0–3	404	10	49	6	< 0.001	453	10
Implementation score	4–6	1708	44	233	31		1941	41
	7–8	915	23	271	36		1185	25
	9–10	895	23	206	27		1101	24
Number of antenatal care visits	0	499	13	12	2	< 0.001	511	11
	1	188	5	9	1		198	4
	2	353	9	44	6		397	9
	3	934	24	174	23		1109	24
	4+	1951	50	518	68		2470	53
Sample size		3926	100.0	758	100.0		4684	100.0

*CBDDM* Community-Based Data for Decision-Making, *HEW* Health extension worker, *SNNP* Southern Nations, Nationalities and Peoples, *WDA* Women’s Development Army

The average treatment effects from PSM models are provided in Table 3. The PSM models were balanced for differences in the co-variates between self-reported exposure status to the Family Conversation strategy (shown in Table 2). The standardized differences in the co-variates between intervention and comparison group respondents before and after matching for the two PSM models (one among all respondents and one among home deliveries) to show their balancing property, are given in an additional file (Additional file 1 for this paper). The average treatment effects on newborn care practices were analyzed among women whose childbirths were not attended by skilled health professionals, while the effects on institutional deliveries and early postnatal care were among all deliveries. The PSM analyses show that exposure to Family Conversations had statistically significant (*p* < 0.05) associations with higher institutional delivery, early postnatal care, clean cord care for the newborn and thermal care for the newborn. The treatment effect for thermal care of the newborn showed the highest effect 16 percentage-points (*p* = 0.001), followed by the effect for early postnatal care (12 percentage-points). The effect for immediate breastfeeding was not statistically significant (*p* = 0.494).

**Table 3:** Maternal and newborn indicators by Family Conversation exposure status and ATEs, matched sample

	Family Conversation		# of matches(α)	Average treatment effect		
	Not exposed	Exposed		%-points	95% Confidence interval	*p*-value
	% (*N*)	% (*N*)				
Institutional delivery	56 (3502)	63 (688)	1–3	7	(1.5, 11.9)	0.011
Early postnatal care	8 (3502)	20 (688)	1–3	12	(7.6, 17.2)	< 0.001
Clean cord care	34 (1622)	48 (207)	7–8	9	(1.3, 16.6)	0.022
Thermal care	40 (1622)	56 (207)	7–8	16	(6.6, 24.7)	0.001
Immediate initiation of breastfeeding	66 (1622)	69 (207)	7–8	3	(−5.4, 11.2)	0.494

(α) Number of comparison area individuals matched per intervention area individual for the propensity score matching models

*ATEs* Average treatment effects

## Discussion

In this study we found additional evidence of the positive impact of Family Conversations on the uptake of certain recommended maternal and newborn health care practices. We found a positive association with institutional delivery, early postnatal care, clean cord care and thermal care for the newborn, suggesting Family Conversations successfully overcame some of the barriers to carry out recommended practices before the intervention program was implemented. These findings are in agreement with similar studies in low and middle-income settings evaluating interventions of increased male involvement during pregnancy [32–35]. The successful improvement of institutional delivery is also in line with other African studies. For example, a study in Tanzania reported that training a pregnant woman, her husband and family members on home-based life skills, through community health workers, improved the involvement of men in accompanying their wives for antenatal care and child birth; in sharing decision-making power on place of delivery and in improving their knowledge of danger signs during pregnancy, delivery and the postpartum period [33].

The effect of Family Conversation on postnatal care visits within 48 h of delivery, clean cord care practices and thermal care of the newborn were striking. These findings are in agreement with another Ethiopian study done in 51 kebeles which reported that having two to four family meetings during pregnancy increased the completeness of care elements during pregnancy, labor and the early postpartum period by 151% [34]. Another similar small scale study with a community-based collaborative approach that involved family meetings, a labor and birth notification system and community quality improvement teams reported increased early postnatal coverage, within 48 h, from 11 to 49% over a two-year intervention period [35]. That our finding is lower, could be related to low exposure time to the intervention, as the data were collected 6 months after initiation of the intervention. In addition, the Family Conversation intervention was implemented in a larger geographic area, which covered 115 woredas and 3070 kebeles and thus implementation at scale may have an influence on the effects of the intervention. It appears that implementation strength for Family Conversation was strong in Tigray region and exposure of young couples to the intervention was low when compared with older couples. It was reported elsewhere in similar study areas that exposure of young married adolescent to Health Extension Program front-line workers was low, compared with older groups [36]. The participation of grandmothers and mothers-in-law in the conversation sessions was relatively low when compared with the participation of husbands, which happened in 80% of the conversation sessions. Had there been high participation of grandmothers, there could have been stronger effects of the intervention.

Literature in other low and middle-income counties in Sub-Saharan Africa and Asia has shown that home visits by community health workers during pregnancy and the early postpartum period, and engagement with women’s groups, contributed to improvements in key behaviors such as skilled birth and newborn care practices [37–41]. Our study, which measures the effect of the Family Conversation intervention on intermediate maternal and newborn health outcomes, is in agreement with studies in other low-income countries.

Ethiopian traditions prescribe that the mother and newborn stay at home during the first 40 days of a newborn’s life, to facilitate rest and recovery [42]. It is usually the task of more senior family members (such as mothers and sisters-in-law) to care for the mother and newborn at home, giving the newly delivered mother and her husband a relatively limited say in the care of the newborn. The cultural practices related to Ethiopian traditions may include immediate bathing of the newborn with cold water, discarding the colostrum, pre-lacteal feeding, applying butter or dung to the cord and restricting the movement of the mother and newborn [43–45]. Our results show that although the coverage of the intervention was low with a short exposure time, in the majority of cases, husbands attended Family Conversations at their home. In most maternal and newborn health care programs, health workers meet and provide counseling to women during pregnancy. Even though their approval and decision is critical in seeking maternal and newborn health care services, husbands are not usually engaged in routine maternal and newborn health-related communication interventions [19]. Family Conversations create an opportunity to engage those household decision makers to help improve care seeking practices in maternal and newborn health care.

Studies in some Sub-Saharan African countries showed that decision-making regarding access to and use of skilled maternal health care services was strongly influenced by preferences and opinions of mothers-in-law and other family members [15, 46, 47] and community-based interventions that engaged grandmothers were associated with improved maternal and child feeding knowledge and practices [48, 49].

### Limitations of the study

The characteristics of the study participants were significantly different between those who reported exposure to Family Conversation and those who did not. PSM has gained popularity to estimate intervention effects in such cases [50] . We thus applied PSM models for estimating intervention effects. The analysis of the balancing property indicated that the covariate patterns were significantly different between exposure statuses. The exposure to the intervention participants who did not have individuals with a similar covariate pattern in the non-exposed participants were excluded in the PSM analysis. In comparison, regression analysis would not have excluded such cases. Thus, we preferred to use the PSM method instead of regression methods.

There were several limitations to the study. First and foremost, selection and recall bias could have over or under-estimated the treatment effects. Treatment effects were potentially over-estimated if the study participants who were more health conscious (and therefore more likely to have better maternal and newborn health care practices) were more likely to recall exposure to Family Conversations, more likely to agree to participate in a Family Conversation, or both. However, the intervention effects could be under-estimated if the WDA or the HEWs targeted Family Conversations towards women who were less likely to have positive maternal and newborn health care practices. We applied a PSM technique to reduce the effects of selection bias. The model controlled for number of antenatal care visits and for the newborn care indicators, it also controlled for early postnatal care, which would reduce the selection bias due to health consciousness. Nonetheless, recall bias would still remain a threat to the validity of this study. Second, the intervention effects could be confounded by the influence of other maternal and newborn health interventions that were not accounted for by the PSM analysis. And third, average treatment effects estimated the effectiveness of the intervention, but did not assess population-level impact. Data collection was conducted only 6 months after initiation of the intervention, so at least half the study participants (women who had a child aged 0–11 months prior to the data collection) could not have been exposed to the intervention.

## Conclusions

Family Conversations were shown to have a potentially positive effect on MNH practices. This could lead to a reduction of maternal and newborn morbidities and mortalities in Ethiopia. With many maternal and newborn care practices higher in the group of participants that had a Family Conversation, the study results suggest that, despite strongly embedded cultural and traditional practices, Family Conversations could potentially play a crucial role in changing a family’s behaviors towards better maternal and newborn care practices and should be further explored. It will be essential to evaluate how to implement this strategy logistically within Ethiopia’s health system, particularly considering the workload of HEWs and the WDA.

## Additional file

Additional file 1: Standardized mean differences of the independent variables between exposed and not exposed to Family Conversation. A table of standardized differences in the co-variates between intervention and comparison group respondents before and after matching for the two PSM models (one among all respondents and one among home deliveries), to show their balancing property. (DOCX 15 kb)

## Abbreviations

CBDDM: Community-Based Data for Decision-Making
HEW: Health Extension Worker
L10 K: Last Ten Kilometers
MNH: Maternal and newborn health
PSM: Propensity score matching
WDA: Women’s Development Army

## References

[1] Central Statistical Agency, Ethiopia. Ethiopia Demographic and Health Survey 2011. Addis Ababa: CSA & ICF International; 2011. https://dhsprogram.com/pubs/pdf/FR255/FR255.pdf. (Accessed 5 May, 2017).

[2] Central Statistical Agency (CSA) [Ethiopia] (2016). Demographic and Health Survey 2016: Key indicators report.

[3] Ronsmans C, Graham WJ (2006). Maternal mortality: who, when, where, and why. Lancet.

[4] Lawn JE, Cousens S, Zupan J. 4 million neonatal deaths: When? Where? Why? Lancet. 2005;365:891–900. 10.1016/S0140-6736(05)71048-5 15752534

[5] Pattinson RS, Kerber K, Buchmann E, Friberg IK, Belizan M, Lansky S (2011). Stillbirths: how can health systems deliver for mothers and babies?. Lancet.

[6] Bhutta ZA, Das JK, Bahl R, Lawn JE, Salam RA, Paul VK (2014). Can available interventions end preventable deaths in mothers, newborn babies, and stillbirths, and at what cost?. Lancet.

[7] Darmstadt GL, Walker N, Lawn JE, Bhutta ZA, Haws RA, Cousens S (2008). Saving newborn lives in Asia and Africa: cost and impact of phased scale-up of interventions within the continuum of care. Health Policy Plan.

[8] Lawn JE, Kerber K, Enweronu-laryea C, Massee O (2009). Newborn survival in low resource settings — are we delivering?. BJOG.

[9] Schiffman J, Darmstadt GL, Agarwal S, Baqui AH (2010). Community-based intervention packages for improving perinatal health in developing countries: a review of the evidence. Semin Perinatol.

[10] Salasibew MM, Filteau S, Marchant T (2014). A qualitative study exploring newborn care behaviours after home births in rural Ethiopia: implications for adoption of essential interventions for saving newborn lives. BMC Pregnancy Childbirth..

[11] Warren C. Care seeking for maternal health: challenges remain for poor women. Ethiopia J Health Dev. 2010;24(1):100–4.

[12] Yargawa J, Leonardi-Bee J (2015). Male involvement and maternal health outcomes: systematic review and meta-analysis. J Epidemiol Community Heal.

[13] Lailulo YA, Susuman AS, Blignaut R (2015). Correlates of gender characteristics, health and empowerment of women in Ethiopia. BMC Womens Health.

[14] Iganus R, Hill Z, Manzi F, Bee M, Amare Y, Shamba D (2015). Roles and responsibilities in newborn care in four African sites. Trop Med Int Heal.

[15] White D, Dynes M, Rubardt M, Sissoko K, Stephenson R (2013). The influence of intra familial power on maternal health care in Mali: perspectives of women, men and mothers-in-law. Int Perspect Sex Reprod Health.

[16] Tekelab T, Yadecha B, Alemu SM (2015). Antenatal care and women’s decision making power as determinants of institutional delivery in rural area of western Ethiopia. BMC Res Notes.

[17] Woldemicael G, Tenkorang EY (2010). Women’s autonomy and maternal health-seeking behavior in Ethiopia. Matern Child Heal J.

[18] Nigatu D, Gebremariam A, Abera M, Setegn T, Deribe K (2014). Factors associated with women’s autonomy regarding maternal and child health care utilization in bale zone: a community based cross-sectional study. BMC Womens Health.

[19] Biratu B, Lindstrom D (2007). The influence of husbands’ approval on women’s use of prenatal care: results from Yirgalem and Jimma towns, south West Ethiopia. Ethiop J Heal Dev.

[20] USAID/MCHIP (2012). Cultural Barriers to Seeking Maternal Health Care in Ethiopia: A Review of the Literature.

[21] Amare Y (2014). Umbilical cord care in Ethiopia and implications for behavioral change: a qualitative study. BMC Int Health Hum Rights.

[22] Mitchell K, Mathewos B, Russell J, Bekele A, Schellenberg J (2016). Tradition and innovation: a qualitative study of changing maternal and newborn health practices in Ethiopia. J Epidemiol Community Health.

[23] Damtew ZA, Karim AM, Willey B, Tesfaye C, Fesseha Zemichael N, Yeshanew B, et al. Correlates of the Women’s Development Army strategy implementation strength with household reproductive, maternal, newborn and child healthcare practices: a cross-sectional study in four regions of Ethiopia. BMC Pregnancy Childbirth. 2018;18(Suppl 1) 10.1186/s12884-018-1975-y. 10.1186/s12884-018-1975-y PMC6157249 30255789

[24] Karim AM, Fesseha Zemichael N, Shigute T, Emaway Altaye D, Dagnew S, Solomon F, et al. Effects of a community-based data for decision-making intervention on maternal and newborn health care practices in Ethiopia: a dose-response study. BMC Pregnancy Childbirth. 2018;18(Suppl 1) 10.1186/s12884-018-1976-x. 10.1186/s12884-018-1976-x PMC6157194 30255793

[25] Wereta T, Betemariam W, Karim AM, Fesseha Zemichael N, Dagnew S, Wanboru A, et al. Effects of a participatory community quality improvement strategy on improving household and provider health care behaviors and practices: a propensity score analysis. BMC Pregnancy Childbirth. 2018;18(Suppl 1) 10.1186/s12884-018-1977-9. 10.1186/s12884-018-1977-9 PMC6157250 30255783

[26] Lemeshow S, Robinson D (1985). Surveys to measure programme coverage and impact: a review of the methodology used by the expanded programme on immunization. World Heal Stat Q.

[27] Filmer D, Pritchett L (2001). Estimating wealth effects without expenditure data – or tears: an application to educational enrollment in states of India. Demography.

[28] StataCorp (2015). Stata: Release 14. Statistical Software.

[29] StataCorp (2015). Stata treatment-effects reference manual: potential outcomes/counterfactual outcomes, release 14.

[30] Austin PC (2009). Balance diagnostics for comparing the distribution of baseline covariates between treatment groups in propensity-score matched samples. Stat Med.

[31] Rosenbaum P, Rubin D (1985). Constructing a control group using multivariate matched sampling methods that incorporate the propensity score. Am Stat.

[32] Aguiar Carolina, Jennings Larissa (2015). Impact of Male Partner Antenatal Accompaniment on Perinatal Health Outcomes in Developing Countries: A Systematic Literature Review. Maternal and Child Health Journal.

[33] August F, Pembe AB, Mpembeni R, Axemo P, August F, Pembe AB, et al. Community health workers can improve male involvement in maternal health : evidence from rural Tanzania Community health workers can improve male involvement in maternal health: evidence from rural Tanzania. 2017;9716

[34] Barry D, Frew AH, Mohammed H, Desta BF, Tadesse L, Aklilu Y (2014). The effect of community maternal and newborn health family meetings on type of birth attendant and completeness of maternal and newborn care received during birth and the early postnatal period in rural Ethiopia. J Midwifery Womens Health.

[35] Tesfaye Solomon, Barry Danika, Gobezayehu Abebe Gebremariam, Frew Aynalem Hailemichael, Stover Kim Ethier, Tessema Hana, Alamineh Lamesgin, Sibley Lynn M. (2014). Improving Coverage of Postnatal Care in Rural Ethiopia Using A Community-based, Collaborative Quality Improvement Approach. Journal of Midwifery & Women's Health.

[36] Karim AM, Tamire A, Medhanyie AA, Betemariam W (2015). Changes in equity of maternal, newborn, and child health care practices in 115 districts of rural Ethiopia: implications for the health extension program. BMC Pregnancy Childbirth.

[37] Gogia S, Sachdev HPS (2016). Home-based neonatal care by community health workers for preventing mortality in neonates in low- and middle-income countries: a systematic review. Nat Publ Group.

[38] Hanson C, Kujala S, Waiswa P, Schellenberg J (2017). Community-based approaches for neonatal survival: meta-analyses of randomized trial data.

[39] Kirkwood BR, Manu A, Asbroek AHA, Soremekun S, Weobong B, Gyan T (2013). Eff ect of the Newhints home-visits intervention on neonatal mortality rate and care practices in Ghana: a cluster randomised controlled trial. Lancet.

[40] Prost A, Colbourn T, Seward N, Azad K, Coomarasamy A, Copas A (2013). Women’ s groups practising participatory learning and action to improve maternal and newborn health in low-resource settings: a systematic review and meta-analysis. Lancet.

[41] Bhutta ZA, Memon ZA, Soofi S, Salat S, Martines J (2008). Implementing community-based perinatal care: results from a pilot study in rural Pakistan.

[42] Warren C (2005). Care of the newborn: community perceptions and health seeking behavior. Ethiop J Heal Dev.

[43] Amare Y, Degefie T, Mulligan B (2012). Newborn care seeking practices in central and southern Ethiopia and implications for community based programming. Ethiop J Heal Dev.

[44] Amare Y, Shamba DD, Manzi F, Bee MH, Omotara BA, Iganus RB (2015). Current Neonatal Skin Care Practices in Four African Sites. J. Trop. Pediatr.

[45] Ganle JK, Obeng B, Segbefia AY, et al. How intra-familial decision-making affects women's access to, and use of maternal healthcare services in Ghana: a qualitative study. BMC Pregnancy Childbirth. 2015;15:173. 10.1186/s12884-015-0590-4 PMC4537557 26276165

[46] Ganle JK, Obeng B, Segbefia AY, Mwinyuri V, Yeboah JY, Baatiema L (2015). How intra-familial decision-making affects women’s access to, and use of maternal healthcare services in Ghana: a qualitative study. BMC Pregnancy Childbirth.

[47] Speizer IS, Story WT, Singh K (2014). Factors associated with institutional delivery in Ghana: the role of decision-making autonomy and community norms. BMC Pregnancy Childbirth.

[48] Mukuria AG, Martin SL, Egondi T, Bingham A, Thuita FM (2016). Role of social support in improving infant feeding practices in western Kenya: a quasi-experimental study. Glob Heal Sci Pract.

[49] Aubel J, Touré I, Diagne M (2004). Senegalese grandmothers promote improved maternal and child nutrition practices: the guardians of tradition are not averse to change. Soc Sci Med Pergamon.

[50] Caliendo M, Kopeinig (2008). Some practical guidance for the implementation of propensity score matching. J Econ Survey.

