# Supplementary material for: Effects of family conversation on health care practices in Ethiopia: a propensity score matched analysis
Source: BMC Pregnancy Childbirth. 2018 Sep 24;18(Suppl 1):372. doi: 10.1186/s12884-018-1978-8 (PMC6157286; doi:10.1186/s12884-018-1978-8)
Supplement: Supplementary file 1 — Standardized mean differences of the independent variables between exposed and not exposed to Family Conversation. A table of standardized differences in the co-variates between intervention and comparison group respondents before and after matching for the two PSM models (one among all respondents and one among home deliveries), to show their balancing property. (DOCX 15 kb) [file 12884_2018_1978_MOESM1_ESM.docx]

| **Table 1c: Standardized mean differences of the independent variables between exposed and not exposed to Family Conversation** | | | | | | | |
| --- | --- | --- | --- | --- | --- | --- | --- |
| Independent variables | |  | Sample with all deliveries | |  | Not attended by skilled health professional | |
|  |  |  | Raw | Matched |  | Raw | Matched |
| Number of ANC visits | | None |  |  |  |  |  |
|  | | 1 | -0.140 | -0.042 |  |  |  |
|  | | 2 | -0.062 | -0.022 |  |  |  |
|  | | 3 | -0.058 | 0.034 |  |  |  |
|  | | 4+ | 0.328 | -0.034 |  |  |  |
| Number of ANC visits | | (continuous) |  |  |  | 0.584 | 0.092 |
| Early PNC | | No |  |  |  |  |  |
|  | | Yes |  |  |  | 0.440 | -0.015 |
| WDA density | | (continuous) | -0.279 | 0.005 |  | -0.455 | -0.028 |
| Age | | (continuous) | 0.182 | 0.023 |  | 0.134 | 0.050 |
| Education | | (continuous) | 0.101 | 0.035 |  | 0.103 | -0.041 |
| Married | | No |  |  |  |  |  |
|  | | Yes | 0.020 | -0.058 |  | 0.020 | -0.033 |
| Number of children | | (continuous) | 0.135 | 0.026 |  | 0.136 | 0.007 |
| Religion | | Orthodox |  |  |  |  |  |
|  | | Protestant | -0.209 | -0.016 |  | -0.318 | -0.088 |
|  | | Muslim | -0.127 | -0.020 |  | -0.236 | 0.003 |
|  | | Other | -0.069 | -0.045 |  | -0.036 | -0.079 |
| Distance to any health facility | | <30 minutes |  |  |  |  |  |
|  | | 30 min - <1 hr | -0.035 | 0.046 |  | -0.043 | -0.024 |
|  | | 1-<2 hrs. | -0.082 | -0.003 |  | -0.135 | -0.016 |
| HEW density | | 2,499 or less |  |  |  |  |  |
|  | | 1:2500-3500 | 0.011 | 0.047 |  | -0.044 | -0.004 |
|  | | 1:3500-5000 | 0.035 | 0.038 |  | 0.121 | -0.078 |
|  | | 1:5000+ | -0.001 | -0.079 |  | -0.169 | -0.056 |
| Wealth quintile | | Lowest |  |  |  |  |  |
|  | | Second | 0.071 | 0.014 |  | 0.137 | -0.081 |
|  | | Middle | -0.047 | 0.020 |  | -0.157 | -0.017 |
|  | | Fourth | -0.056 | -0.052 |  | -0.052 | 0.020 |
|  | | Highest | 0.058 | 0.046 |  | -0.031 | 0.088 |
| Region | | Tigray |  |  |  |  |  |
|  | | Amhara | -0.038 | 0.059 |  | 0.171 | 0.051 |
|  | | Oromia | -0.195 | -0.077 |  | -0.188 | -0.066 |
|  | | SNNP | -0.128 | -0.003 |  | -0.262 | 0.012 |
| CBDDM implementation strength | | (continuous) | 0.251 | 0.064 |  | 0.131 | -0.065 |
| Raw and Matched denote ‘before’ and 'after’ matching analyzed by propensity score matching analysis (indicator variables were used for categorical measures) | | | | | | |  |
